# Liquid–Liquid Phase Separation Induced by Vapor Transfer in Evaporative Binary Sessile Droplets

**DOI:** 10.1021/acs.langmuir.3c01686

**Published:** 2023-09-07

**Authors:** Ahmed M. Othman, Andreas. S. Poulos, Ophelie Torres, Alexander. F. Routh

**Affiliations:** † Department of Chemical Engineering and Biotechnology, University of Cambridge, Philippa Fawcett Dr, Cambridge CB3 0AS, U.K.; ‡ Unilever R & D Port Sunlight, Quarry Road East, Wirral CH63 3JW, U.K.

## Abstract

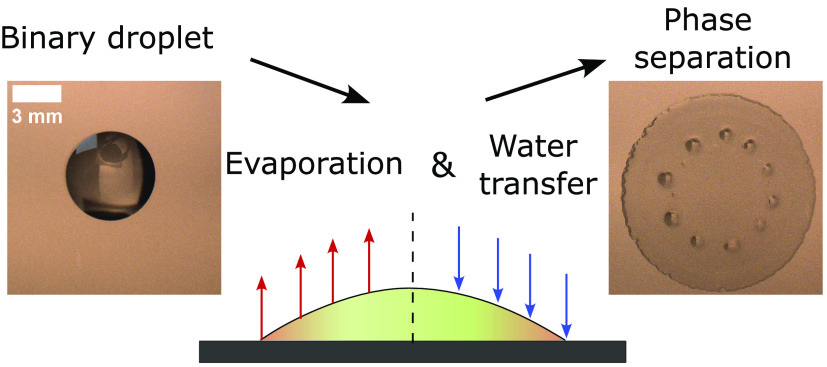

Drying of binary sessile droplets consisting of ethanol and octamethyltrisiloxane on a high-energy surface is investigated. During the process of evaporation, the droplets undergo liquid–liquid phase separation, resulting in the appearance of microdroplets at the liquid–air interface, which subsequently violently burst. This phase separation is attributed to water vapor transfer into the droplet, which modifies the solubility and leads to the formation of a ternary mixture. The newly formed ternary mixture may undergo nucleation and growth or spinodal decomposition, depending on the droplet composition path. By control of the relative humidity of air, phase separation can be mitigated or even eliminated. The droplets also display high mobility and complex wetting behavior due to phase separation, with two contracting and two spreading stages. The mass loss experiments reveal that the droplets undergo three distinct drying stages with an enhanced evaporation rate observed during the phase separation stage. A modified diffusion-limited model was employed to predict the evaporation rate, accounting for the physiochemical changes during evaporation and proved to be consistent with experimental observations. The findings of this work enhance our understanding of a coupled fundamental process involving the evaporation of multicomponent mixtures, wetting, and phase separation.

## Introduction

The evaporation of a sessile droplet constitutes a fundamental process with significant industrial applications, including but not limited to inkjet printing and fabrication of micro/nanoelectronics,^1,2^ insecticides,^3^ crime scene reconstruction,^4^ and DNA microarrays.^5,6^ The evaporation of single-component droplets on a solid substrate has been extensively studied and is well understood, with numerous publications examining this phenomenon.^7−10^

The dynamics of evaporation in multicomponent droplets, containing two or more liquids, give rise to increased complexity owing to selective evaporation, surface tension gradients, and thermal effects.^11−14^ These factors influence the wetting dynamics and contact angle during evaporation.^15−17^ Baumgartner et al.^18^ demonstrated that droplets containing a fully miscible ternary mixture (water, ethanol, and propylene glycol), deposited on completely wetting surfaces, exhibit rich wetting dynamics as selective evaporation and surface tension gradients can change the droplet shape and result in three different wetting behaviors. Furthermore, the aforementioned factors in multicomponent droplet evaporation also play a crucial role in understanding deposit patterns when polymers or particles are included.^19−21^ Deposit patterns, for instance, a coffee ring or multiple deposits due to stick and slip motion of the droplet, are influenced by surface tension gradients, wetting dynamics, composition of the binary mixture, and evaporation dynamics.^22−24^

The evaporation dynamics of a droplet can be significantly influenced by external environmental factors as they may facilitate water condensation and/or absorption during evaporation. Such alterations may lead to changes in the evaporation rate of the droplet.^25,26^ This has been experimentally demonstrated through the application of time-resolved infrared spectroscopy to ethanol droplet evaporation, where it was observed that water absorption can create a binary droplet with distinctive evaporation dynamics.^27^ Evaporation of pure ethanol and ethanol–water droplets in various relative humidity conditions was investigated by Ozturk and Erbil.^28^ The authors reported that the increase in relative humidity corresponds to an increase in water vapor adsorption into droplets. The evaporation rate of pure ethanol at high relative humidity was reported to decrease after some time, and a slower evaporation rate was observed, with a value similar to the evaporation rate of pure water. The authors reported similar observations for a binary mixture containing ethanol and water.^28^ Kita et al.^25^ and Chen et al.^26^ also reported analogous findings, where the increase in relative humidity around a pure ethanol droplet leads to the formation of an ethanol–water mixture. Gas injection chromatography was used to measure the concentration of water absorbed into droplets.^25^

The process of multicomponent sessile droplet evaporation is susceptible to further intricacies, such as the occurrence of phase separation.^14,29−33^ The solubility of two compounds in a mixture depends on several factors, including composition, temperature, pressure, and the nature of the components. The demixing of a homogeneous liquid mixture can be induced by changes in temperature.^34^ Evaporative cooling coupled with compositional changes during evaporation of partially miscible *n*-hexane and 2-(2-ethoxyethoxy)ethanol can induce phase separation.^32^ Recently, Chao et al.^33^ investigated the effect of phase separation during the evaporation of water and different glycol ethers, such as di(propylene glycol) propyl ether on a heated surface. They observed a strong correlation among phase separation, contact line dynamics, and evaporation dynamics. The droplets undergoing phase separation on a fully wetting surface exhibited sudden spreading, which did not follow the classic Cox–Voinov law.^33^

Selective evaporation has been identified as a means of inducing demixing in multicomponent mixtures.^14,29,30,35^ The initial composition of a binary or ternary mixture can determine the mechanism of the liquid–liquid phase separation. The mixture separates via spontaneous emulsification, forming microdroplets of similar size, a phenomenon commonly referred to as the “ouzo effect”.^35−37^ Alternatively, larger liquid sheets separating the two liquid phases can form through spinodal decomposition.^38−40^ The behavior of ternary mixture droplets containing ethanol, water, and anise oil, known as ouzo droplets, is particularly intriguing and complex during the evaporation process.^29^ On a hydrophobic octadecyltrichlorosilane glass surface, an ouzo droplet displays static behavior with a pinned contact line, and the mixture initially appears homogeneous and clear.^29^ However, as the good solvent (ethanol) evaporates, the mixture displays complex flow behavior and begins to phase separate, with the formation of oil microdroplets at the droplet edge.

Another notable phase separation in multicomponent droplets occurs during the evaporation of water droplets containing the nonvolatile 1,2-hexanediol.^30^ The selective evaporation of the volatile component, water, leads to accumulation of the less volatile component, 1,2-hexanediol, at the droplet edge. This ultimately results in insufficient convective and diffusive mixing inside the droplet, leading to phase segregation. Eventually, evaporation of the mixture ceases when the entire surface of the droplet is covered by a layer of 1,2-hexanediol, with water trapped underneath. Phase separation in multicomponent droplets holds significant importance for various industrial applications. For instance, the nanoprecipitation process utilizes phase separation via spontaneous emulsification to produce functional nanomaterials that are used for drug delivery,^37,41,42^ catalysis,^43^ energy,^44^ and cosmetics.^45^ Therefore, gaining a comprehensive understanding and control of the fundamental mechanisms underlying phase separation and evaporation have the potential to improve efficiency in these applications.

In this study, we investigate the wetting behavior, phase separation, and drying dynamics that occur during the evaporation of a binary droplet consisting of octamethyltrisiloxane and ethanol on a smooth glass slide with a high-energy surface. Unlike previous studies, such as ouzo droplets,^29^ which involved a pinned droplet containing three liquids, two of which are immiscible, the binary system investigated in this paper consists of an initially miscible binary mixture where droplets exhibit four different wetting behaviors during the evaporation process. This study will demonstrate that phase separation in this system is triggered by a distinct mechanism that differs from mechanisms described in previous research papers^30,33^ and can be controlled experimentally. Furthermore, this study uncovers the different drying periods that droplets undergo. To the best of our knowledge, evaporation and phase separation of octamethyltrisiloxane and ethanol binary droplets have not been previously investigated. Octamethyltrisiloxane is used in various industrial applications, including personal care products,^46,47^ industrial coatings,^48^ thin-film deposition,^49^ textiles,^50^ and pharmaceutical products.^51^ Ethanol and octamethyltrisiloxane mixtures are commonly used in various personal care products, such as skin creams and deodorants, where ethanol acts as a carrier for the active ingredients, while octamethyltrisiloxane contributes to the texture and feel of the product. Therefore, understanding the evaporation dynamics of this mixture is important for optimizing product stability during formulation and product application under different environmental conditions.

## Experimental Section

### Materials

The droplets used in this study are composed of ethanol (Fisher Scientific, 99.5% Extra Dry, AcroSeal) and octamethyltrisiloxane (Sigma-Aldrich, 98%). The binary mixture was prepared by mixing both solvents at the desired weight concentration using an analytical balance (Sartorius Entris Essential).

### Droplet Visualization and Evaporation Procedure

**Figure 1:**
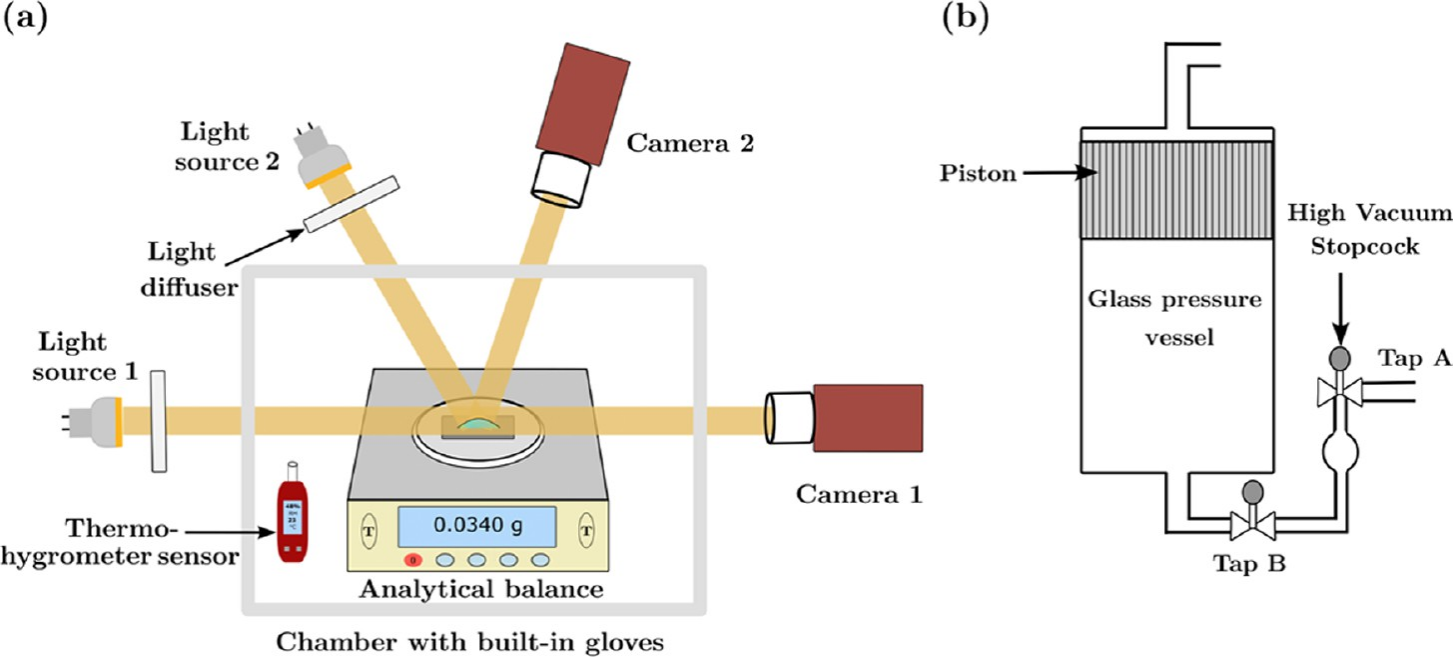
(a) Sketch of the experimental configuration used for droplet visualization and mass loss experiments. Two cameras are positioned to capture top-view and side-view images along with a mass balance to report the mass loss of the droplets. This arrangement is encompassed by an inflatable polyethylene chamber with built-in gloves, facilitating droplet deposition and relative humidity control. (b) Sketch of the custom pressure apparatus used to measure the vapor–liquid equilibrium (VLE) of octamethyltrisiloxane and ethanol binary mixtures at 23 ± 0.5 °C.

A 5 μL binary droplet with different initial octamethyltrisiloxane concentrations, ranging from 10 to 70 wt %, was deposited on a smooth glass slide (Fisher Scientific, SuperFrost) using an Eppendorf micropipet (1–20 μL) with an error tolerance of 0.3%. This procedure was conducted within a controlled environment provided by an enclosed inflatable polyethylene chamber equipped with built-in gloves (Aldrich, AtmosBag). The relative humidity within the chamber was maintained at a specific level by using a saturated salt solution of potassium hydroxide (Sigma-Aldrich). Additionally, a data logger (RS Components) was employed to record the temperature and humidity conditions inside the chamber. The relative humidity ranged from 20 to 50% RH, and the temperature was maintained within a range of 22–23 °C. The evolution of the binary droplet over time was observed through two charge-coupled device (CCD) cameras (FLIR, EOS-Flea 3) capable of capturing 60 frames per second. One of the cameras was positioned to capture top-view images, and the other was designated to capture side-view images. The resulting top-view images were analyzed using ImageJ software to determine the droplet radius, while the side-view images were analyzed using OpenDrop image analysis software to quantify the droplet contact angle.^52^ Additionally, the droplet mass loss during drying was recorded simultaneously by employing an analytical balance (Sartorius Entris Essential) with a measurement error of 0.1 mg. The analytical balance and cameras were connected to a computer to record the droplet mass loss, radius, and contact angle simultaneously. The experimental setup used in this study is depicted in Figure 1a. To capture images and videos of the region near the droplet contact line, an optical microscope (Leica DME) equipped with a 10× working objective lens (working distance = 12 mm, numerical aperture = 0.25) was employed. The optical microscope was equipped with a Brunel microscope camera (L3CMOS, 60 fps).

### Properties of the Binary Mixture

A custom-designed pendant droplet setup was employed to measure the surface tension of the mixture within a closed environment, containing ethanol vapor to mitigate evaporation-related effects. The acquired images from the experimental setup were analyzed using OpenDrop software. The vapor–liquid equilibrium (VLE) of the binary mixture was determined using a specialized pressure vacuum apparatus shown in Figure 1b. The apparatus was partially immersed in a 23 ± 0.5 °C thermostated water bath. A vacuum pump was connected to both ends of the apparatus. A mixture containing octamethyltrisiloxane and ethanol with a total volume between 3 and 3.5 mL was added from tap A and allowed to vaporize into the large volume of the apparatus, which was under vacuum, by opening tap B. The vapor pressure of the mixture was measured using a manometer (RS components, RS PRO absolute and differential manometers) connected to the apparatus via tap A after allowing some time for the mixture to reach equilibrium. The liquid composition was separated from the vapor by tilting the apparatus forward to accumulate the liquid between taps A and B, and the vapor composition was compressed using the apparatus piston. A detailed description of this methodology can be found in ref (53). The vapor and liquid concentrations at equilibrium were measured using a Bellingham refractometer (RFM 340). The calibration curve showing refractive index and octamethyltrisiloxane mass fraction is shown in Figure S2a in the Supporting Information (SI).

The impact of temperature changes on the binary mixture during the evaporation process was measured. To achieve this, a 20 mL octamethyltrisiloxane and ethanol mixture was contained in a glass vial with a rubber seal and subjected to slow cooling in a water bath. The mixture was continuously stirred by using a magnetic stirrer, and a thermocouple was inserted to monitor the temperature throughout the process. A CCD camera was also used to detect any phase separation or mixture turbidity that occurred during the temperature reduction.

### Ternary Phase Diagram and Water Vapor Transfer

A ternary phase diagram for a mixture of ethanol, octamethyltrisiloxane, and water was established through titration experiments at a temperature of approximately 23 °C. An octamethyltrisiloxane and ethanol mixture was prepared at a specific concentration, and water was then gradually added to the mixture until the mixture became turbid, indicating the onset of phase separation. The amount of water added to the mixture was measured by using an analytical balance (Sartorius Entris Essential). This process was repeated for different octamethyltrisiloxane and ethanol mixture concentrations, and the data were used to construct the solubility phase diagram.

The rate of water vapor transfer into a pure ethanol droplet was quantified as a function of time at 23 °C and a relative humidity of 49% RH, using a Bellingham refractometer (RFM 340). A 5 μL droplet of pure ethanol was deposited on a smooth, hydrophobic polytetrafluoroethylene surface and allowed to evaporate for a predetermined period. Subsequently, the droplet was transferred to the refractometer using a micropipet, and the amount of water that had diffused into the droplet during the evaporation process was measured. A calibration curve for a binary mixture of water and ethanol was established and found to align with previous studies.^54^ The calibration curve showing the refractive index and water mass fraction is shown in Figure S2b in the SI.

## Results and Discussion

**Figure 2:**
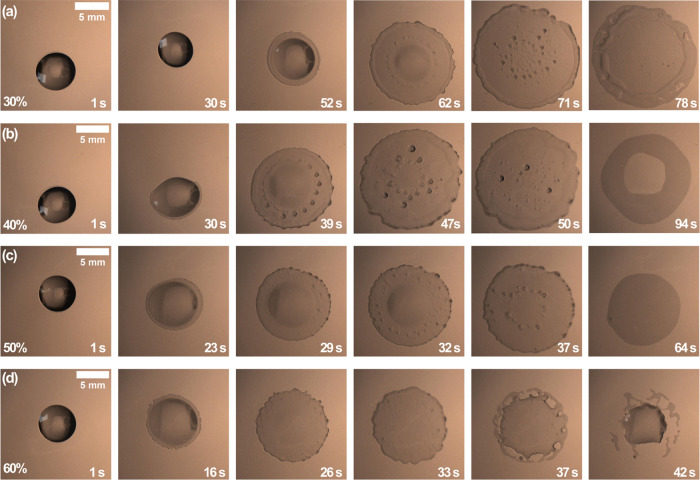
Top-view images of octamethyltrisiloxane and ethanol droplets at different time intervals and initial octamethyltrisiloxane compositions of (a) 30 wt %, (b) 40 wt %, (c) 50 wt %, and (d) 60 wt %. In each sequence, a 5 μL droplet was deposited on a precleaned glass slide at 23 °C and 49% RH.

Droplets with initial octamethyltrisiloxane concentrations in ethanol ranging from 10 to 70 wt % exhibit distinctive dynamics during evaporation, when deposited on a high-energy surface. Figure 2 depicts top-view images of droplets with initial octamethyltrisiloxane concentrations ranging from 30 to 60 wt % on a glass substrate at 23 °C and 49% RH. The complete set of images of the binary droplets investigated in this study covers a range of concentrations from 10 to 70 wt %, as shown in Figure S1 in the SI.

Initially, the droplets in Figure 2 retain a spherical cap shape before rapidly spreading and generating smaller, observable microdroplets that ultimately burst over time. A uniform pattern in the formation of microdroplets in the mixture is substantiated by their appearance at a specific radial distance from the droplet center, as shown in Figures 2a,b at 62 and 39 s, respectively. The concentration of the mixture has a discernible effect on the size of the microdroplets, with a reduction in size apparent at initial concentrations of 60 wt % and higher. Unexpectedly, following the sudden spreading, the droplet radius then decreases and the shape of the droplets shifts to a spherical cap. Eventually, at concentrations exceeding 50 wt %, the contracting droplets begin to spread again, albeit slowly. It is noteworthy that these droplets exhibit high mobility during the early stages after deposition, with the entire droplet moving in a nonuniform manner. The unique dynamics of these binary droplets are shown in Movies S1–S3 in the SI.

### Marangoni Contraction

As soon as the droplet is deposited on the surface, it maintains its shape and displays a finite apparent contact angle, θ_app_, despite complete wetting of the substrate by both individual pure components. This hydrodynamic mechanism is known as Marangoni contraction.^12,13,55^

**Figure 3:**
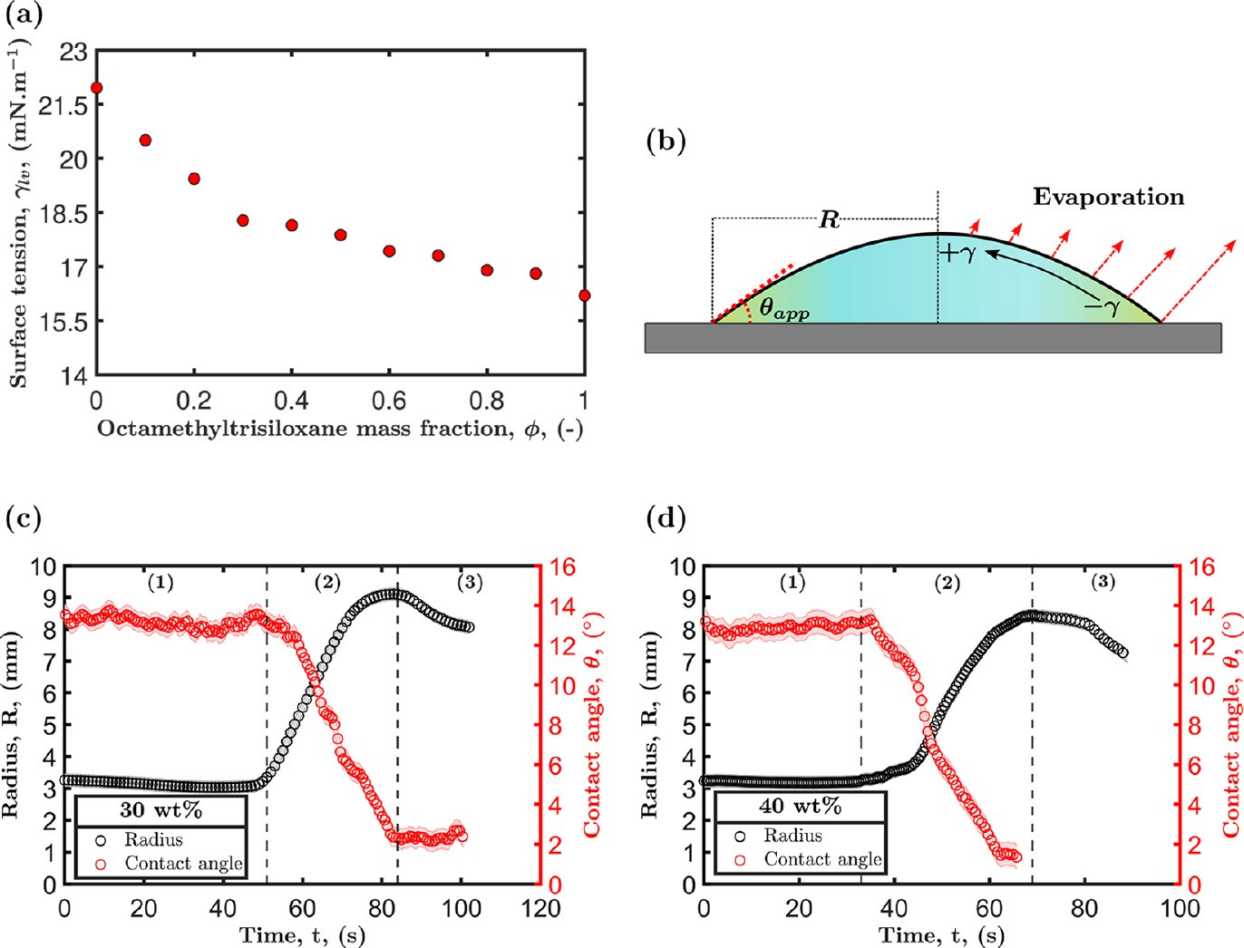
(a) Surface tension γ_*lv*_ of the octamethyltrisiloxane and ethanol binary mixture measured at 23 °C using a pendant drop technique at different octamethyltrisiloxane mass fractions ϕ. (b) Schematic illustrating the surface tension gradient of an evaporated binary droplet with differing volatility and surface tension causing an inward flow and maintaining the shape of the droplet. (c) Droplet radius *R* and contact angle θ_app_ versus time for a 30 wt % octamethyltrisiloxane in ethanol, measured at 23 °C and 49% RH. (d) Droplet radius *R* and contact angle θ_app_ versus time for a 40 wt % octamethyltrisiloxane in ethanol, measured at 23 °C and 49% RH. The vertical lines in panels (c) and (d) represent the different wetting stages that droplets experience during evaporation.

Marangoni contraction can be triggered when the most volatile component in a binary mixture has the highest surface tension.^55^ The binary droplets meet this requirement, with ethanol (the most volatile component) having a surface tension of approximately 22 mN/m (as demonstrated in Figure 3a) and a vapor pressure of 6.9 kPa, while octamethyltrisiloxane exhibits a surface tension of 16.2 mN/m and a vapor pressure of 0.52 kPa. The depletion of ethanol at the droplet edge occurs first due to the nonuniform evaporation across the droplet, with the highest value thought to occur near the contact line.^12,13,19^ This nonspatially uniform evaporation increases the concentration of octamethyltrisiloxane near the contact line and lowers the local surface tension. An alternative explanation is that the reduced height at the droplet edge leads to evaporation having a more pronounced effect on the local concentration.^56^ The compositional change across the droplet leads to an inverted flow from the edge to the center, hindering the outward spreading of the droplet and maintaining the quasi-stationary shape with a nonzero contact angle. This process is sketched in Figure 3b. During this stage, droplets also exhibit high mobility on the substrate (refer to Movies S1, S2, S8, and S9 in the SI), indicating that the contact line is not pinned^33^ and that Marangoni flow might break the axial symmetry of the droplet, possibly resulting in this random mobility.^14^

The temporal change of the radius and apparent contact angle for droplets with initial octamethyltrisiloxane concentrations of 30 and 40 wt % are shown in Figure 3c,3d. During the Marangoni contraction, as indicated by region 1, the apparent contact angle θ_app_ remains approximately constant for both initial concentrations and the radius slightly decreases. The apparent contact angle θ_app_ appears to oscillate slightly, with the value changing up to ±0.5° over time. This small variation can be attributed to the droplet being highly mobile, i.e., not pinned, on a completely wetting surface.

### Mechanistic Understanding of Microdroplet Formation

**Figure 4:**
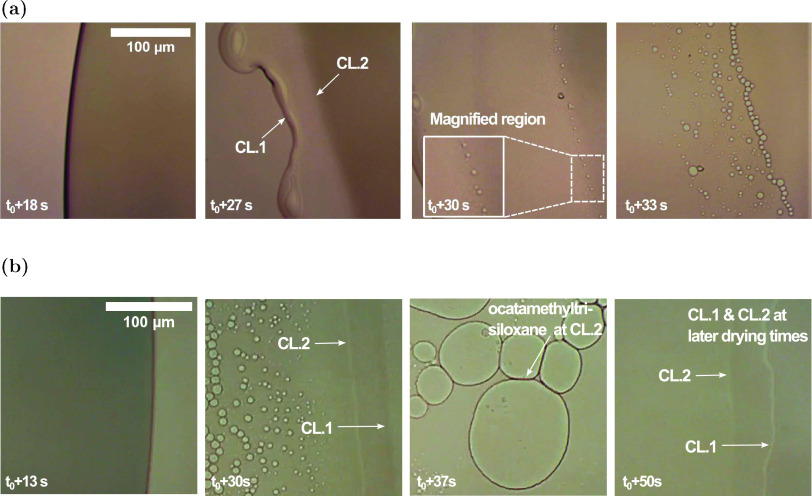
Top-view images of a region near the contact line of two 5 μL droplets imaged using an optical microscope at different time intervals with initial octamethyltrisiloxane compositions of (a) 20 wt % and (b) 50 wt %. The droplets were deposited on a precleaned glass slide at 23 °C and 49% RH. For both compositions, two contact lines are formed (CL.1 and CL.2), and the formation of microdroplets appears in contact line 2, with a separate boundary between the two contact lines.

The second stage is characterized by an instantaneous spreading, soon followed by the formation of microdroplets. This is highlighted as region 2 in Figure 3c,d, where the droplet radius abruptly increases and the contact angle decreases sharply. From the top-view images of the contact line region shown in Figure 4a,b, it appears that the contact line is initially homogeneous, indicating that the mixture is fully miscible during the initial period after deposition. Subsequently, the contact line splits into two visible contact lines, denoted as CL.1 and CL.2. Each contact line exhibits a distinct behavior: CL.1 exhibits an outward spreading motion away from the center of the droplet, while microdroplets start to appear around CL.2. The microdroplets started to coalesce and grew in size at a specific radial distance across the droplet, as shown in Figure 4b. Both contact lines remain separated even later in the evaporation process, as depicted in Figure 4b, before CL.2 completely disappears and CL.1 continues to wet the surface.

#### Isothermal Vapor–Liquid Equilibrium

**Figure 5:**
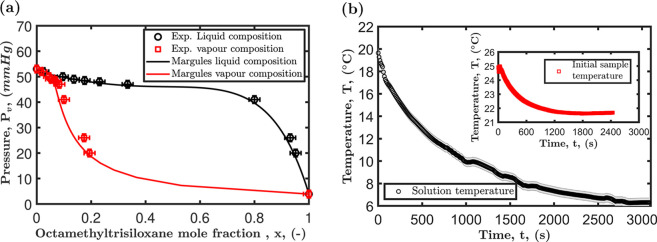
(a) Isothermal VLE diagram of the octamethyltrisiloxane and ethanol mixture measured at 23 ± 0.5 °C. The solid lines denote the fitted Margules activity model, and the data points represent the measured values. (b) Temperature profile of a 20 mL octamethyltrisiloxane and ethanol mixture contained in a glass vial with an initial octamethyltrisiloxane concentration of 30 wt % during the slow cooling process to investigate phase separation. The inset in the figure shows the initial solution temperature stabilizing before the start of the slow cooling process.

The thermodynamic equilibrium between the vapor and liquid phases of the octamethyltrisiloxane and ethanol mixture facilitates determining the bubble point and dew point curves. These curves dictate the conditions under which bubbles and droplets form and persist. The hypothesis that the observed phase separation is due to vapor formation within the mixture may be valid if the VLE diagram shows a negative deviation from Raoult’s law, leading to a minimum in the boiling point of the mixture at a certain concentration range. The isothermal VLE diagram of the mixture is presented in Figure 5a, with the bubble point curve represented by the black data points and the dew point curve represented by the red data points. The Margules activity model is utilized to predict the activity coefficient γ_*i*_ of each component in the mixture. This quantifies the deviation from ideal behavior.^57^

In the concentration range of 0.1 to 0.8, as depicted in Figure 5a, the results demonstrate that the mixture begins to bubble when the total vapor pressure is between 52 and 46 mmHg at 23 °C. However, the drying experiments were performed at atmospheric pressure, i.e., 760 mmHg. This implies that the observed phase separation is not caused by vapor formation within the binary mixture.

#### Liquid–Liquid Phase Separation

Sadafi et al.^32^ showed that liquid–liquid phase separation in binary mixtures might be initiated by the reduction in temperature associated with evaporative cooling. Ethanol is a highly volatile liquid, with a vapor pressure of approximately 6.9 kPa at 23 °C. The evaporation of ethanol results in a phase change from liquid to vapor, removing the latent heat required for the transformation and resulting in a reduction in droplet surface temperature of a magnitude of several degrees Celsius.^16,32^ As a result, microscopic phase separation could arise in this multicomponent mixture as it is subjected to a temperature change during evaporation. Figure 5b depicts the temperature reduction of a 20 mL octamethyltrisiloxane and ethanol mixture contained in a glass vial with an initial octamethyltrisiloxane concentration of 30 wt %. The inset to Figure 5b shows how the temperature of the mixture was stabilized, remaining spatially isothermal at room temperature. It is noted that leaving the initial mixture for 30 min prior to the cooling experiment ensures an initial thermal equilibrium. Despite lowering the temperature to near 6 °C, the mixture remained homogeneous throughout, and there was no evidence of phase separation. These results negate the hypothesis that evaporative cooling can lead to liquid–liquid phase separation in octamethyltrisiloxane and ethanol mixtures.

#### Influence of Relative Humidity on Liquid–Liquid Phase Separation

According to Kita et al.,^25^ the impact of relative humidity on the evaporation of pure ethanol droplets can be substantial. The absorption or condensation of water into pure ethanol droplets creates a binary mixture, altering the evaporation dynamics under varying relative humidities. This could have a significant impact on the binary droplet, as the addition of a third component, water, creates a ternary mixture that can alter the solubility and may result in solution demixing.

**Figure 6:**
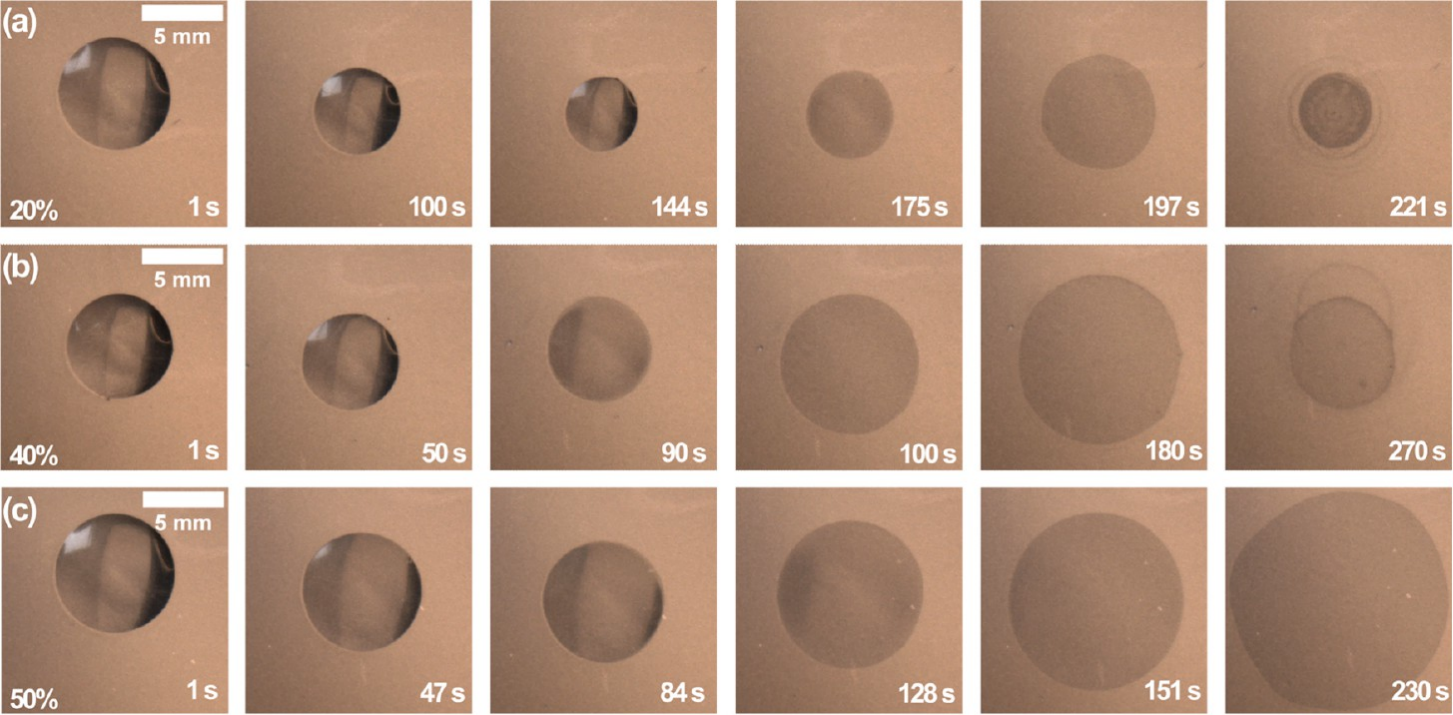
Top-view images captured using a CCD camera of octamethyltrisiloxane and ethanol droplets at different time intervals and compositions deposited on a precleaned glass slide at 23 °C and 21 ± 1% RH. A 5 μL droplet was pipetted on the substrate with different initial octamethyltrisiloxane concentrations of (a) 20 wt %, (b) 40 wt %, and (c) 50 wt %.

Given that octamethyltrisiloxane is soluble in ethanol but not in water,^58^ the amount of water present in the mixture can play a role in determining demixing. The relative humidity in the environment surrounding the evaporated droplet was maintained at approximately 21 ± 1% RH to investigate its effect on phase separation (refer to Figure S3b in the SI). Figure 6 shows top-view images of octamethyltrisiloxane and ethanol droplets with different initial compositions imaged at different time intervals at approximately 21 ± 1% RH and 23 °C.

A crucial observation from Figure 6, as well as Movie 4 and Movie 5 in the SI, is the absence of visible microdroplets, indicating that phase separation within the mixture is suppressed at low relative humidity levels. This suggests that phase separation is likely driven by the transfer of water from the surrounding environment into the droplet, creating a ternary mixture. The amount of water transferred into the binary mixture of octamethyltrisiloxane and ethanol then determines the onset of phase separation.

**Figure 7:**
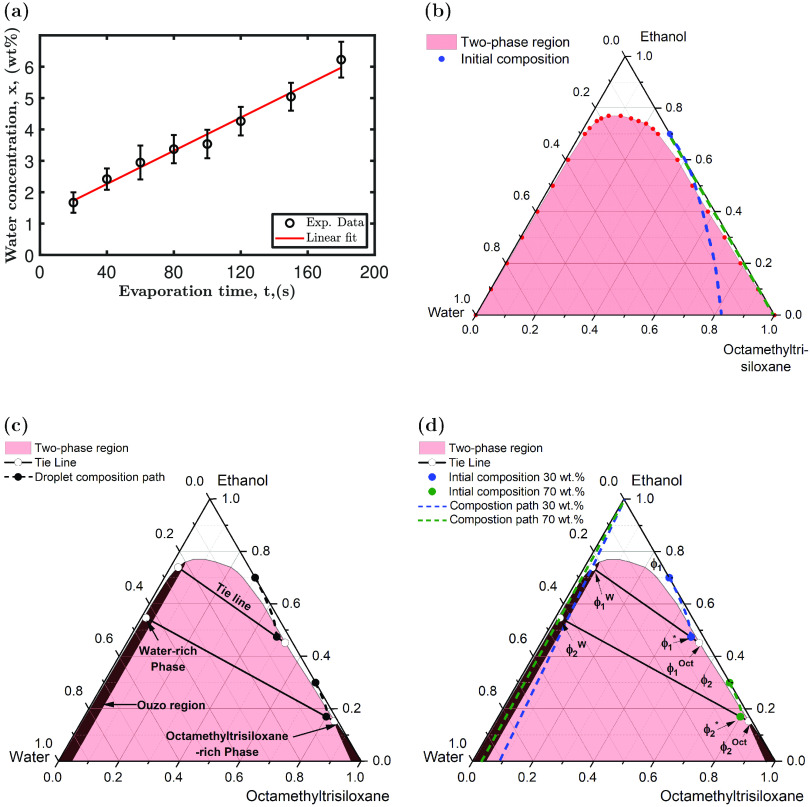
(a) Water vapor concentration as a function of evaporation time of a 5 μL pure ethanol droplet drying at 49% RH and 23 °C. (b) The measured ternary phase diagram of the octamethyltrisiloxane, ethanol, and water system at 23 °C showing the two-phase region highlighted in red. The green line represents the evaporation path when no water transfer occurs, and the blue line represents the evaporation path when there is water transfer into the droplet. (c) Ternary phase diagram of the octamethyltrisiloxane, ethanol, and water system illustrating the two-phase region highlighted in red, tie lines, the ouzo region highlighted in black, and a potential compositional path for 30 and 70 wt % droplets. (d) Ternary Phase diagram showing the compositional paths of ϕ_1_ (30 wt %) and ϕ_2_ (70 wt %) droplets, denoted by blue and green dashed lines, respectively. The resulting two subphases, water-rich and octamethyltrisiloxane-rich, resulting from the liquid–liquid phase, are highlighted for both concentrations and denoted by the superscripts W and Oct.

In the binary droplet, only ethanol is hygroscopic and absorbs water from the atmosphere. Figure 7a depicts that the amount of vapor transfer into a pure ethanol droplet increases with evaporation time at 49% RH. After 20 s of evaporation, the pure ethanol droplet contains approximately 2 wt % of water. This amount increased linearly with time, reaching 6 wt % at an evaporation time of 180 s. The transfer of water vapor into an evaporating droplet may occur as a result of the absorption, adsorption, or condensation of water molecules. Condensation of water vapor on the ethanol droplet is possible due to evaporative cooling at the surface of the droplet, which may fall below the dew point temperature. In our experiments, the dew point temperature was estimated to be approximately 11 °C.^59^ Kita et al.^25^ argues that due to the enhanced evaporation of ethanol droplets at higher relative humidity, condensation is possible. However, in our system, IR thermography and micro-thermocouple measurements of the bulk and interfacial temperatures of pure ethanol droplets have shown that the temperature of the droplet bulk is around 17 °C and 50% RH.^60^ As a result, the likelihood of water transfer via vapor condensation is minimal.

The abundance of water vapor around the droplet has the potential to increase water adsorption and absorption at higher humidity values. The measured amount of water transfer into a pure ethanol droplet, shown in Figure 7a, demonstrates that an octamethyltrisiloxane and ethanol droplet might absorb similar amounts during evaporation. The resulting ternary mixture presents a complex system with potential impacts on the flow dynamics, wetting properties, and evaporation.^29,33^

The measured miscibility phase diagram of a multicomponent system containing octamethyltrisiloxane, ethanol, and water is depicted in Figure 7b, with the shaded area showing the region where two distinct phases exist. The initial concentrations of the droplets investigated in this system are located on the right-hand side axis with an initial water concentration of zero.

Two main processes are simultaneously occurring, i.e., ethanol evaporation and water absorption. The evaporation path of a droplet can be estimated based on the volatility of each component. Figure 7b shows a potential compositional path for a droplet with an initial concentration of 30 wt % octamethyltrisiloxane in ethanol. The composition path is estimated by a straight line along the axis, indicating a decrease in ethanol concentration. The green line in Figure 7b depicts the variation in composition of the mixture in the absence of water transfer into the droplet. However, this scenario changes as the relative humidity rises, with water transferring to the droplet during evaporation. This causes the composition path line to shift toward the water axis, as indicated by the dashed blue line, causing the mixture to undergo a phase separation. Therefore, it can be inferred that the occurrence of liquid–liquid phase separation during the evaporation of octamethyltrisiloxane and ethanol droplets is a consequence of water vapor transfer into the droplet. The modulation of the relative humidity levels around the droplets appears to exert a significant suppressive effect on phase separation.

#### Evaporation Path and Microdroplet Formation Mechanisms

Liquid–liquid phase separation mechanism depends on the evaporation path of the droplets, which could be via nucleation and growth or spinodal decomposition. As shown in Figure 4a,4b, the contact line separates into two with clear boundaries prior to the nucleation of the microdroplets. This indicates that the mixture is located within the two-phase region, and each of the subphases has a unique composition that lies on the binodal curve. After the bulk separates into two subphases, the composition of each subphase can be determined through the use of tie lines, which determine the composition of each subphase. The outer contact line, CL.1, is octamethyltrisiloxane-rich, and CL.2 is water-rich, where phase separation is manifested. The CL.1 phase does not readily evaporate and continues to spread until the end of the droplet’s life, supporting the suggestion that CL.1 is an octamethyltrisiloxane-rich phase. CL.2 is the water-rich phase where the nucleation of microdroplets is manifested. The composition of each subphase is estimated by the compositional path of the mixture.

Figure 7c shows a phase diagram of the ternary mixture, illustrating the two-phase region, ouzo regions, tie lines, and possible compositional paths for 30 and 70 wt % droplets. The composition of the water-rich subphase is crucial in determining phase transition mechanism, i.e., nucleation and growth and/or spinodal decomposition. Furthermore, given the difficulty in accurately quantifying the concentrations of the three components in the mixture during evaporation, Figure 7d presents a possible composition path for two droplets with varying concentrations. The blue data point represents a droplet with an initial concentration ϕ_1_ of 30 wt % octamethyltrisiloxane in ethanol. At a mixture concentration of ϕ_1_^*^, the droplet crosses the two-phase region. The concentration of each phase can be determined by a tie line with a water-rich subphase ϕ_1_^w^ and an octamethyltrisiloxane-rich subphase ϕ_1_^oct^.

**Figure 8:**
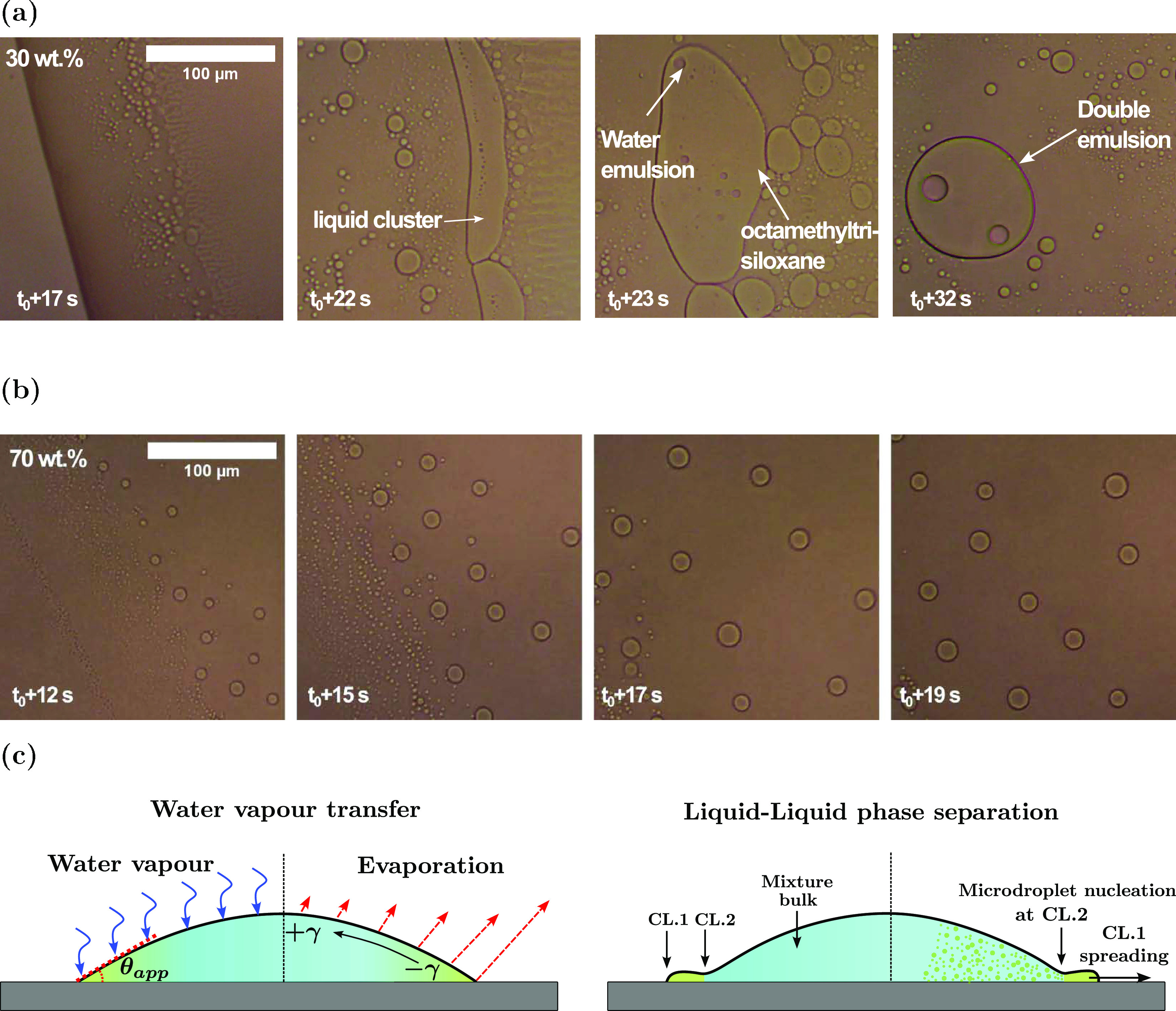
Optical microscope images depict the two mechanisms of liquid–liquid phase separation for two 5 μL droplets with initial octamethyltrisiloxane concentrations: (a) 30 and (b) 70 wt %. (c) Sketch illustrating the second stage that octamethyltrisiloxane and ethanol droplets undergo during evaporation. The sketch shows two consecutive mechanisms: the transfer of water vapor into the droplet, followed by liquid–liquid phase separation inducing droplet spreading.

As ethanol continues to evaporate from each subphase, the composition of the water-rich subphase ϕ_1_^w^ initially enters the ouzo region, leading to the nucleation of small droplets of octamethyltrisiloxane. The composition at CL.2, ϕ_1_^w^, changes further due to ethanol evaporation, as approximated in Figure 7d by the straight blue dashed line passing through ϕ_1_^w^. This mixture may undergo spinodal decomposition by forming larger liquid sheets or clusters as it re-enters the two-phase region. This scenario is in agreement with the experimental observations, as shown in Figure 8a and Movie S6 in the SI. The formation of liquid clusters and agglomerates after nucleation and growth of octamethyltrisiloxane microdroplets suggests that the mixture is at the spinodal limit during later stages of ethanol evaporation. Eventually, these liquid clusters coalesce into larger droplets that burst violently. The violent bursting, observed in Figure 2, is attributed to the buoyancy of octamethyltrisiloxane within the water-rich subphase.

The transition from nucleation and growth to spinodal decomposition is observed in concentrations between 30 and 50 wt %, which explains the violent bursting as larger agglomerates form within this range. In addition, when ϕ_1_^w^ at CL.2 undergoes spinodal decomposition, double emulsions are formed with water starting to nucleate inside the larger octamethyltrisiloxane droplets, as depicted in Figure 8a. This suggests that the octamethyltrisiloxane-rich agglomerate at CL.2 is located in the inverse ouzo region at the bottom-right corner of the ternary diagram in Figure 7d.

The phase separation was observed to change with droplets at higher octamethyltrisiloxane concentrations. The violent bursting due to phase separation is significantly weakened, as Figure 2 indicates. Droplets with concentrations between 60 and 70 wt % undergo a different phase transition. The green data points in Figure 7d depict a proposed phase transition route for a droplet containing 70 wt % octamethyltrisiloxane in ethanol. At the early stages of phase separation, the behavior is similar to droplets containing 30 to 50 wt % of octamethyltrisiloxane. The droplets cross the binodal curve, resulting in the formation of two subphases, i.e., ϕ_2_^w^ and ϕ_2_^oct^. Following the composition path of the water-rich subphase, indicated by the green dashed line passing by ϕ_2_^w^ in Figure 7d, this subphase is maintained within the ouzo region during the entire evaporation process. Therefore, a nucleation and growth mechanism controls the phase separation in the water-rich subphase. The optical images for a 70 wt % octamethyltrisiloxane droplet in Figure 8b and Movie S7 support this argument. This phase separation mechanism results in the formation of smaller and more uniform microdroplets. The average size of the microdroplets emerging during phase separation via nucleation and growth is approximately 14 ± 2 μm; therefore, bursting is less vigorous compared to larger droplets formed via spinodal decomposition with an average diameter of approximately 200 ± 30 μm.

### Spreading Induced by Liquid–Liquid Phase Separation

At the onset of phase separation, CL.1 starts to spread, as shown in region 2 of Figure 3c,d. The observed spreading at CL.1 is due to the predominantly octamethyltrisiloxane composition, which preferentially wets the glass surface.^33^ Suppression of phase separation by reducing the relative humidity eliminates abrupt spreading, as shown in Figure 6. Therefore, the sudden spreading in region 2 is attributed to liquid–liquid phase separation during droplet drying under high relative humidity conditions. Figure 8c is a sketch that illustrates the second stage observed during the evaporation of octamethyltrisiloxane and ethanol droplets.

### Second Marangoni Contraction

**Figure 9:**
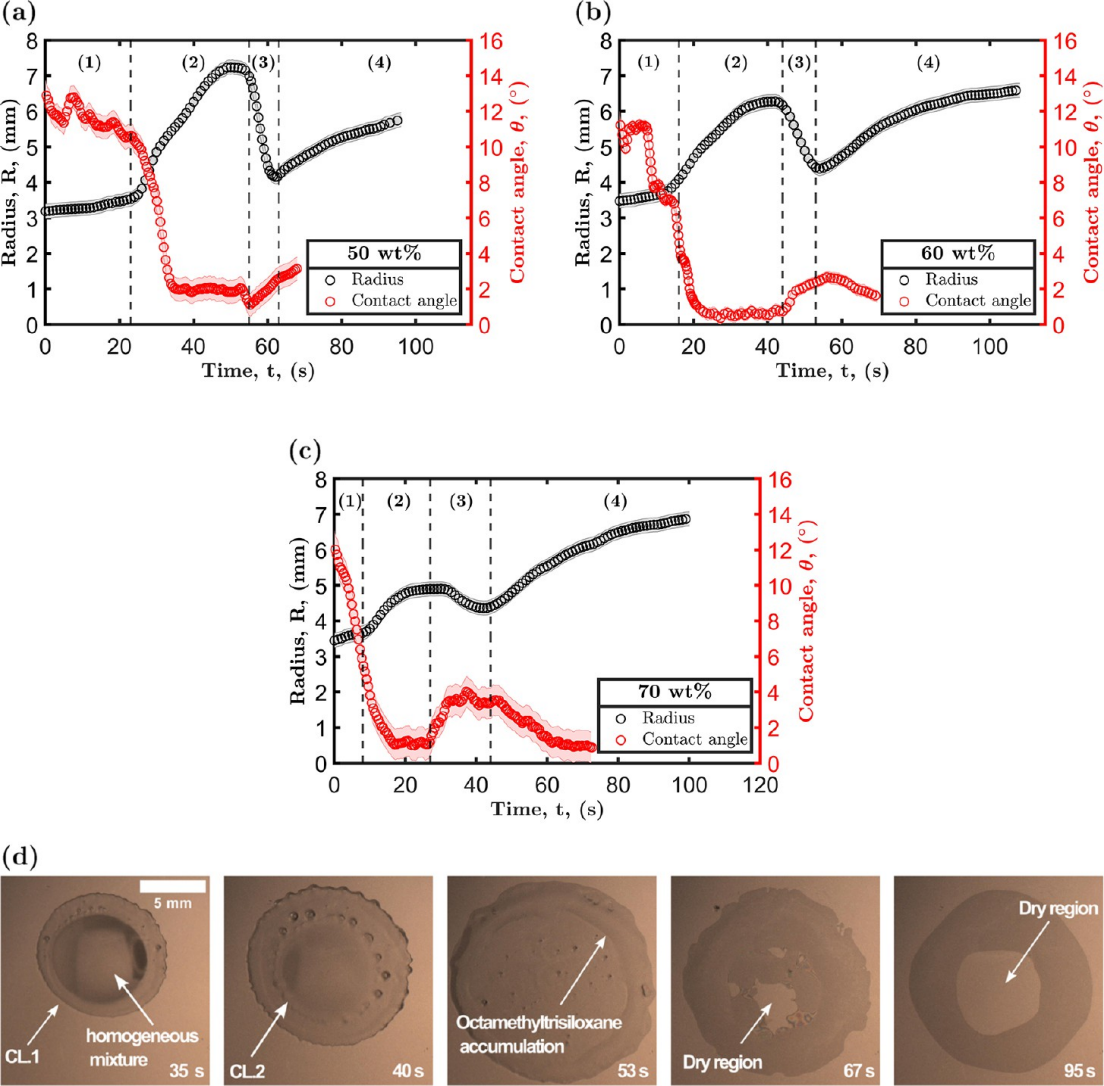
Droplet radius *R* and contact angle θ_*app*_ during evaporation of octamethyltrisiloxane and ethanol droplets with three initial octamethyltrisiloxane concentrations measured at 23 °C and 49% RH: (a) 50 wt %, (b) 60 wt %, and (c) 70 wt %.The vertical lines in panels (a–c) represent the different wetting stages that droplets experience during evaporation. (d) Images showing a droplet initially containing 40 wt % octamethyltrisiloxane in ethanol, illustrating the distribution of octamethyltrisiloxane during drying at 23 °C and 49% RH.

Following liquid–liquid phase separation, droplets with higher initial octamethyltrisiloxane concentrations display another significant contraction. This is demonstrated by examining the wetting of droplets and the apparent contact angle, as shown in Figure 9a–c, for octamethyltrisiloxane concentrations ranging from 50 to 70 wt %.

Figure 9a–c presents the four stages that the droplet radius and contact angle undergo during evaporation. In region 1, droplets show a slow increase in radius, but the droplets maintain their shape. This was followed by rapid spreading in region 2 as a result of liquid–liquid phase separation. Then, there was a sharp contraction in region 3 with a later further spreading in region 4. In this range of octamethyltrisiloxane initial concentrations, the radius and the contact angle in regions 1 and 2 behave similarly to droplets with initial octamethyltrisiloxane concentrations of 20 to 40 wt % as shown in Figure 3.

The second contraction, denoted as region 3 in Figure 9a–c, is distinguished by a sharp decrease in the droplet radius. The occurrence of this stage can also be seen very briefly at concentrations between 30 and 40 wt % in Figure 3c,3d. This sudden contraction in the droplet radius indicates an inward flow from the edge to the center, attributed to the existence of a concentration gradient caused by liquid–liquid phase separation. The two separate phases resulting from liquid–liquid phase separation contain different concentrations of octamethyltrisiloxane. The subphase with a lower octamethyltrisiloxane concentration at CL.2 nucleates into smaller droplets, while the octamethyltrisiloxane-rich subphase at CL.1 spreads outward. Upon completion of phase separation, the distribution of octamethyltrisiloxane across the droplet is altered, leading to a higher concentration at the edge and a corresponding surface tension gradient and inward flow. The octamethyltrisiloxane can be identified from optical images, as it does not evaporate as readily as ethanol, as shown in Figure 9d. Figure 9d displays dry regions at the droplet center after 67 s. This demonstrates the concentration inhomogeneity caused by the phase separation. In addition, it has been shown that liquid–liquid phase separation can produce a concentration gradient between the separated phases,^61,62^ which can lead to region 3 observed in our system.

### Spreading of Pure Octamethyltrisiloxane

This is the last stage that droplets undergo, and it is denoted as region 4 in Figure 9a–c. Upon complete evaporation of ethanol, the residual solvent within the droplet is identified as the least volatile compound, i.e., octamethyltrisiloxane. The contraction of the droplet results in a higher contact angle, and pure octamethyltrisiloxane begins to wet the surface. The analysis of the droplet’s refractive index during this stage shows that the composition of the droplet is entirely octamethyltrisiloxane.

**Figure 10:**
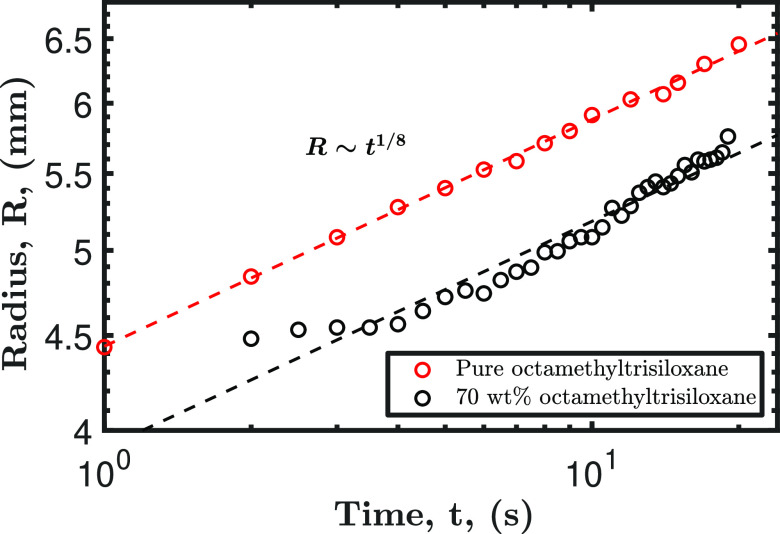
Droplet radius vs time for two different droplets. The black data points represent the spreading behavior of a 5 μL droplet during the fourth stage of drying, initially containing 70 wt % of octamethyltrisiloxane. The red data points depict the spreading dynamics of a 5 μL droplet of pure octamethyltrisiloxane droplet.

The droplet follows the characteristics of a viscous droplet that completely wets a surface. As shown by the black data points in Figure 10, the fourth stage of the droplet’s evolution is described by a power law, specifically Tanner’s law, with the radius scaling as either t^1/8^ or t^1/10^, depending on the droplet’s size.^63,64^ This is compared to the spreading behavior of a pure octamethyltrisiloxane droplet, as represented by the red data points in Figure 10, which closely match the fourth stage of the 70 wt %.

## Evaporation Rate and Drying Periods

**Figure 11:**
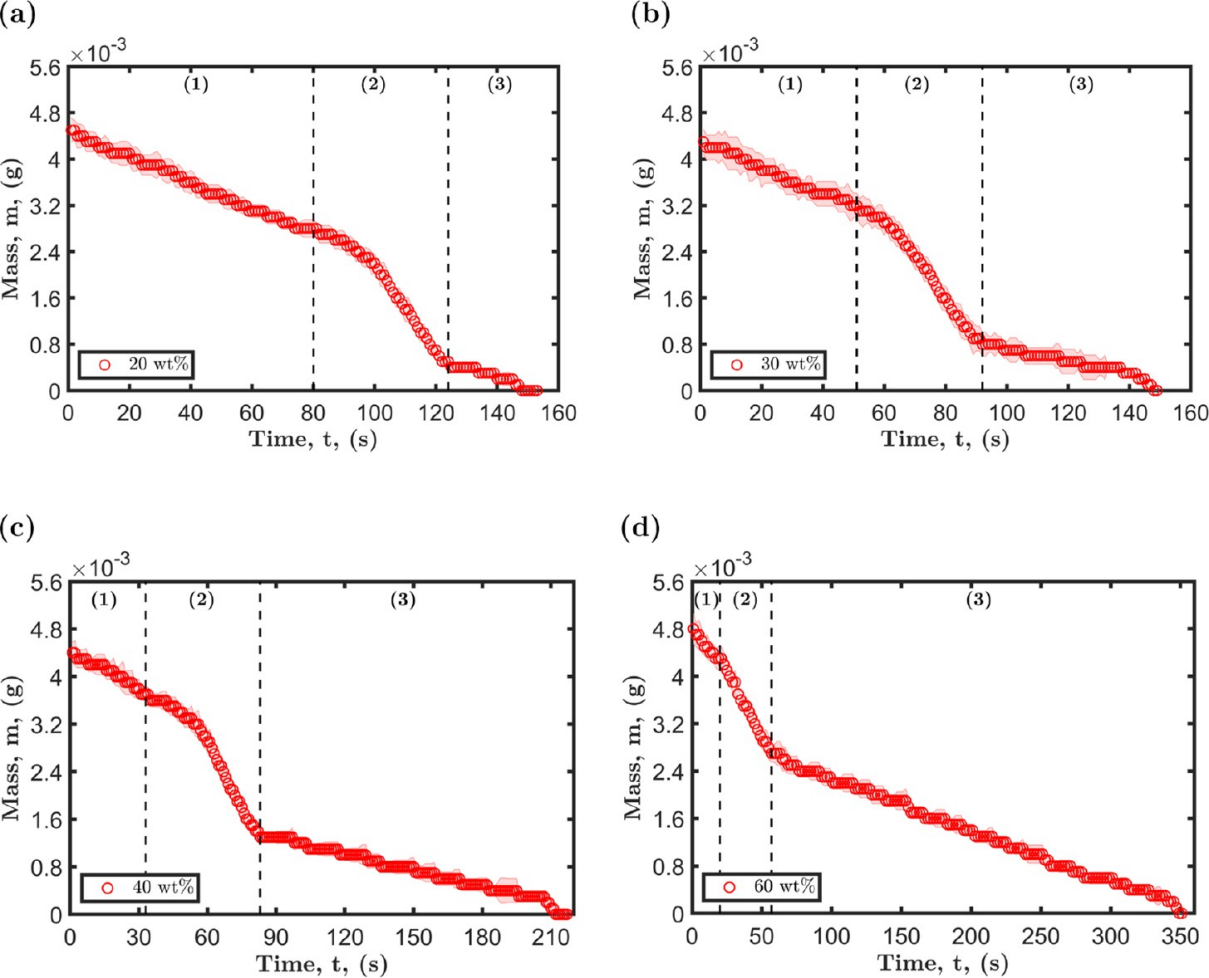
Mass loss curves for droplets containing octamethyltrisiloxane and ethanol dried at 23 °C and 49% with initial octamethyltrisiloxane concentrations: (a) 20 wt %, (b) 30 wt %, (c) 40 wt %, and (d) 60 wt %. The horizontal dashed line serves as a visual representation of the distinct stages of the drying process.

The decrease in the mass of a 5 μL droplet with time is monitored for various concentrations of octamethyltrisiloxane in ethanol, and the results are presented in Figure 11. The mass loss curves depict three main drying periods, which are directly related to the four stages of the droplet’s evolution described in the preceding sections. The first drying period depicted in Figure 11 is representative of the Marangoni contraction stage, i.e., region 1. During this stage, the rate of evaporation is primarily dominated by the partial vapor pressure of ethanol. Subsequently, the slope of the drying curve experiences a significant change as the droplet transitions into the second drying stage, which is characterized by liquid–liquid phase separation and second Marangoni contraction, i.e., regions 2 and 3. The substantial increase in mass loss observed during the second drying period can be attributed to the sudden spreading associated with liquid–liquid phase separation. As the radius of the droplet expands, the rate of evaporation increases, resulting in a corresponding increase in mass loss.^7,8,65^ Additionally, the homogeneity of the mixture is lost as two distinct liquids form, leading to each liquid exerting its own saturated vapor pressure.^57^ This, in turn, increases the rate of mass loss. At the end of the second drying period, a significant reduction in the rate of mass loss is observed. During this period, evaporation is primarily dominated by a less volatile component. The duration of each drying period is influenced by the relative concentration of the components in the mixture.

### Multicomponent Modified Diffusion-Limited Model

Evaporation in this multicomponent system is more intricate than in a single-component system; both systems are governed by diffusion of vapor into the surrounding air. The majority of previous studies focused on diffusion of saturated vapor above the droplet as the sole rate-limiting factor, ignoring the influence of convective air currents.^7,16,66^ Experiments were conducted in a sealed environment to eliminate the effects of air currents. Despite the potential impact of water transfer into the droplet, resulting in liquid–liquid phase separation, its contribution to the evaporation rate is disregarded. The justification for this is that the amount of water is small, as is evident from Figure 7a.

In the work of Popov,^7^ an expression was presented to characterize the evaporation process of a pure sessile droplet as
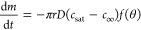
1where
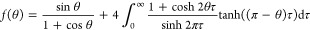
2

Here, *m* is the mass of the droplet, *r* is the droplet radius, *c*_sat_ is the saturated concentration above the droplet, *c*_∞_ is the concentration far away from the droplet, and *f*(θ) is a function of the contact angle.

This expression considers that the evaporation process is in a quasi-steady state. This is due to the time required for vapor concentration surrounding the droplet to adjust to changes in the droplet’s shape being significantly shorter than the overall timescale of the droplet evaporation process.^8^Equation 1 was modified to predict the evaporation rate of binary droplets composed of ethanol and water.^16^ The prediction is based on the mutual contribution of the vapor concentration of both components as well as the average diffusivity of the vapor of both components in air.

Sudden changes in the radius occur due to liquid–liquid phase separation, leading to an increase in the radius by up to a factor of 3. These abrupt modifications in the droplet could invalidate the assumption that the diffusion-controlled evaporation process is in a quasi-steady state. We estimate the time required for the vapor of the octamethyltrisiloxane and ethanol system to adjust to changes in the droplet shape as *R*^2^/*D*. The evaporation time *t*_f_ is given by *R*^2^ρ/*D*(*c*_s_ – *c*_∞_), where ρ is the droplet density. Hence, the ratio of times can be estimated as *R*^2^/*Dt*_f_ ≈ *c*_s_ – *c*_∞_/ρ. For the binary droplets, *c*_s_ ≈ 10^–4^ g/cm^3^, *c*_∞_ = 0 g/cm^3^, and ρ ≈ 0.8 g/cm^3^; hence, *c*_s_ – *c*_∞_/ρ ≈ 1.25 × 10^–4^ ≪ 1. It can be concluded that the time for the vapor phase to adapt to the geometry changes is small, indicating that evaporation of the binary droplets can be considered quasi-steady.

### First Drying Period

It is possible to modify eq 1 in order to accommodate the different drying periods depicted in Figure 11. During the initial stage of evaporation, the droplet is considered homogeneous, and the total vapor pressure can be found by summing up the individual contributions of each constituent. The nonideal behavior of the binary mixture can be accounted for by incorporating the activity coefficients, which can be obtained from the VLE diagram in Figure 5a. The total vapor pressure of the mixture, which governs the total evaporation rate, is *P*_Total_ = *P*_e_ + *P*_*o*_ = *x*_e_ γ_e_*P*_e_^sat^ + *x*_*o*_ γ_*o*_*P*_o_^sat^, where subscripts *e* and *o* refer to ethanol and octamethyltrisiloxane, *P*^sat^ is the saturated vapor pressure at 23 °C, *x* is the liquid mole fraction, and γ is the activity coefficient.

The large difference between the vapor pressures of ethanol and octamethyltrisiloxane results in the total evaporation rate in the first drying period being primarily governed by the diffusive flux of ethanol. In addition, the VLE diagram in Figure 5a indicates that the contribution of octamethyltrisiloxane to the total pressure is negligible, with the total pressure being approximately equal to the partial vapor pressure of ethanol, i.e., *P*_total_ ≈ *x*_e_ γ_e_*P*_e_^sat^. This approximation holds true until the liquid composition reaches a mole fraction of octamethyltrisiloxane of 0.7 or a mass fraction of 0.92. Consequently, the contribution of octamethyltrisiloxane can be disregarded during the initial stage of the drying process. Therefore, it is possible to modify the pure evaporation rate expression, as presented in eq 1, to account for the evaporation rate during the initial drying period for our binary mixture as follows
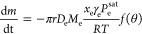
3where *R* is the gas constant and *T* is the temperature. The diffusion coefficient of ethanol is *D*_e_ ≈ 14 × 10^–6^ m^2^·s^–1^ at 23 °C.^67^ The partial vapor pressure of ethanol, *x*_e_ γ_e_*P*_e_^sat^, is found using Figure 5a, which exhibits slight variations based on the initial concentration of the mixture in question. The partial vapor pressure expression, i.e., *x*_e_ γ_e_*P*_e_^sat^, of the various droplet concentrations is tabulated in Table 1. The radius in eq 3 is the measured value from Figures 3c,d and 9a–c.

**Table 1:** Ethanol Partial Vapor Pressure of Different Initial Octamethyltrisiloxane Concentrations during the First Drying Period^a^

droplet initial concentration	partial vapor pressure
ϕ	*x*_e_ γ_e_*P*_e_^sat^
(wt %)	(kPa)
20	6.64
30	6.48
40	6.31
60	5.96

a The values are extracted from Figure 5a.

### Second Drying Period

Upon initiation of phase separation, which is observed by the increase in the droplet radius, two immiscible liquids are formed. These newly formed mixtures are independent, and each separately undergoes evaporation.^57^ The emergence of octamethyltrisiloxane microdroplets can be observed during phase separation, and they rise to the droplet–air interface. Therefore, each component exerts its saturated vapor pressure, with the total vapor pressure equal to the sum of both saturation vapor pressures, i.e., *P*_total_ ≈ *P*_e_^sat^ + *P*_o_^sat^. Equation 1 can be reformulated to adopt this stage as follows
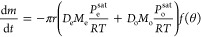
4

The estimation of the diffusion coefficient of octamethyltrisiloxane in air is carried out using the Fuller–Schettler–Giddings equation (refer to the SI). This equation is commonly used to predict the diffusivity of nonpolar components in air and has an estimated error of 5.4%.^59^ The diffusion coefficient of octamethyltrisiloxane is estimated to be 6.41 × 10^–6^ m^2^·s^–1^. The saturated vapor pressures for both pure ethanol and octamethyltrisiloxane at 23 °C are measured to be approximately equal to 6.9 and 0.52 kPa.

### Final Drying Period

During the final drying period, the droplet vapor pressure will be equal to the saturation vapor pressure of octamethyltrisiloxane. Hence, the rate of evaporation can be expressed using eq 1 as
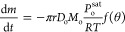
5

The evaporation rate and corresponding mass loss during the drying process can be predicted through the use of eqs 3–5. Since the mass, radius, and contact angle have been recorded simultaneously, the transition time between each individual drying stage is precisely determined from the changes in the droplet radius.

**Figure 12:**
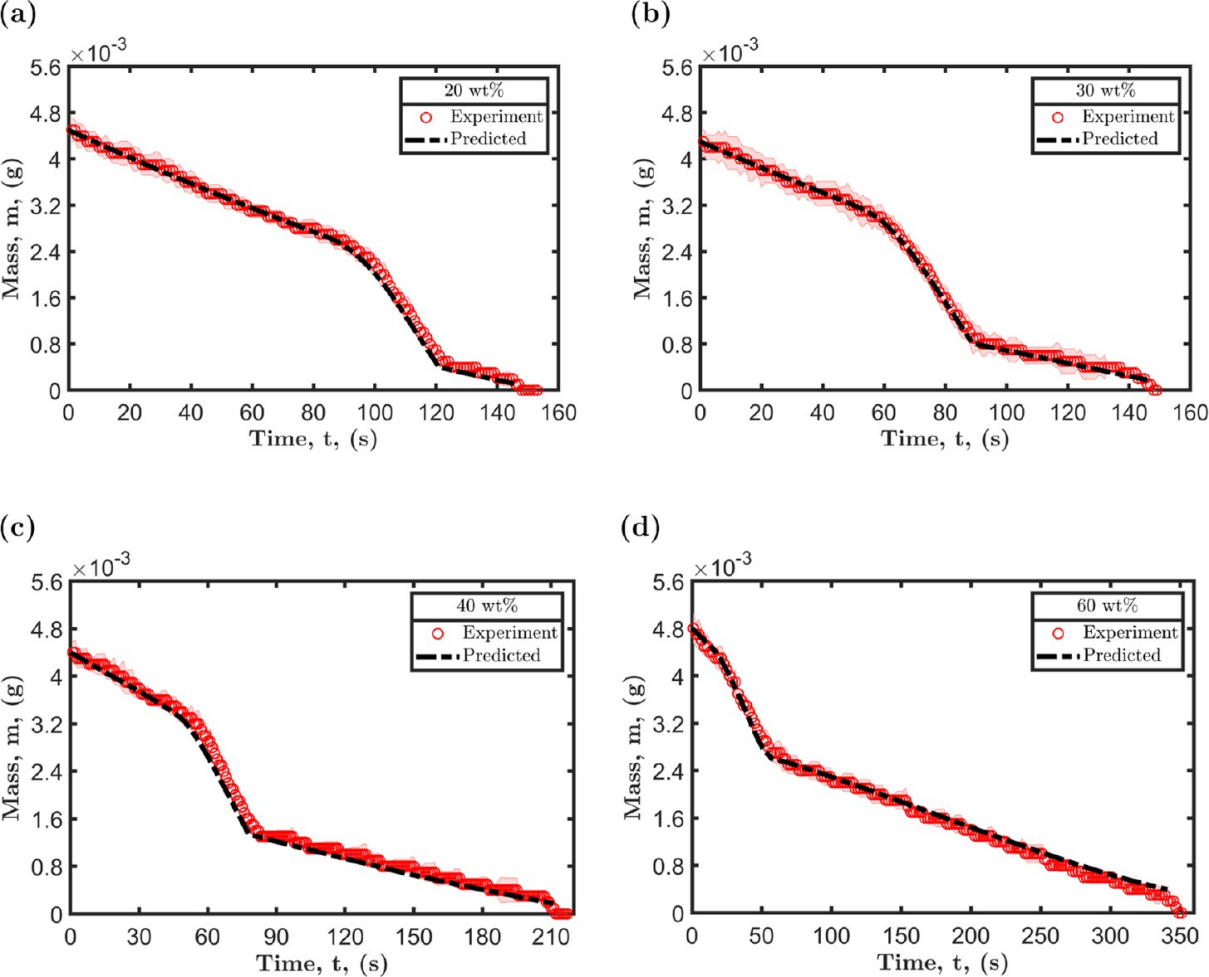
Comparison between the experimental mass loss curves, represented by red circles, and the predicted mass loss, represented by the black dashed line, for octamethyltrisiloxane and ethanol droplets with initial octamethyltrisiloxane concentrations: (a) 20 wt %, (b) 30 wt %, (c) 40 wt %, and (d) 60 wt %.

As depicted in Figure 12, the predicted mass loss for the three stages of drying aligns very well with the experimental measurements. The first stage of drying is in close agreement with the experimental data, with an error margin ranging from 2 to 4% for all concentrations. However, for the second and third stages, there is a deviation of 5–10% between the predicted and experimental values. This discrepancy can be attributed to several factors such as the imprecision in determining the transition and the estimation of the diffusion coefficients. Despite these limitations, the results demonstrate that the modified evaporation rate adapted for each of the stages can make accurate predictions.

The modified model proposed here captures the overall experimentally measured evaporation rate. In the mixture investigated in this study, the components exhibit significantly distinct vapor pressures, enabling the consideration of individual drying stages. Nonetheless, models based on the lubrication approximation will be required when the mixtures components have similar vapor pressures to predict the temporal changes in mixture composition.^18,29,30,68^

## Conclusions

This study investigates the evaporation of droplets containing octamethyltrisiloxane and ethanol on a wettable surface. The findings indicate that the droplets exhibited four distinct wetting dynamics: two contracting and two spreading. A violent bursting of the newly formed microdroplets has been observed within the droplet domain. It was concluded that the observed microdroplets at the vapor–liquid interface were due to liquid–liquid phase separation. The initial mixture of octamethyltrisiloxane and ethanol appeared homogeneous, but during the drying process, the mixture underwent spontaneous phase separation, resulting in the formation of either microdroplets or larger agglomerates depending on the underlying phase separation mechanism. The temperature of the mixture had no effect on the phase separation, but the hygroscopic nature of pure ethanol caused water vapor transfer into the droplet, impacting solubility and resulting in the observed phenomenon. The potential evaporation path was explored for the droplets by using the phase diagram of the ternary mixture, which aided in understanding the phase separation mechanisms. We have experimentally shown that the phase separation is reduced or even suppressed by controlling the humidity of the environment surrounding the droplet. We also investigated the mass loss of the binary droplets, which were found to have three distinct drying periods with different evaporation rates. The vapor–liquid equilibrium of the mixture was determined, and a modified diffusion-limited evaporation model for each drying period was used to predict the mass loss of the droplets. The predicted evaporation rate showed excellent agreement with the experimental evaporation rates. The findings of this work have potential implications for a wide range of coating applications including agriculture, food, inkjet printing, and cosmetics. Implementing this understanding could lead to homogeneous film deposition in the formation of polymer films through multicomponent solvent drying. This can be achieved by controlling system properties, such as relative volatility, air humidity, wettability, and solubility. Furthermore, incorporating spontaneous emulsification caused by solvent evaporation in a multicomponent system during the formation of polymer thin films can result in the formation of microdroplets. Currently, this technique is being explored for slow-release applications.

## Supporting Information Available

Further experimental images, calibration curves, diffusivity estimation, and drying conditions (PDF)
Movie S1: Movie for a droplet with an initial octamethyltrisiloxane concentration of 30 wt % in ethano (MP4); Movie S2: Movie for a droplet with an initial octamethyltrisiloxane concentration of 50 wt % in ethanol (MP4); Movie S3: Movie for a droplet with an initial octamethyltrisiloxane concentration of 60 wt % in ethanol (MP4); Movie S4: Movie for a droplet with an initial octamethyltrisiloxane concentration of 30 wt % in ethanol in 21% RH (MP4); Movie S5: Movie for a droplet with an initial octamethyltrisiloxane concentration of 50 wt % in ethanol in 21% RH (MP4); Movie S6: Movie for a droplet contact line region with an initial concentration of 40 wt % (MP4); Movie S7: Movie for a droplet contact line region with an initial concentration of 70 wt % (MP4); Movie S8: Movie for side-view video for a droplet with an initial concentration of 30 wt % (MP4); Movie S9: Movie for side-view video for a droplet with an initial concentration of 50 wt % (MP4) (ZIP)
