# Supplementary material for: Liquid–Liquid Phase Separation Induced by Vapor Transfer in Evaporative Binary Sessile Droplets
Source: Langmuir. 2023 Sep 7;39(37):13242–57. doi: 10.1021/acs.langmuir.3c01686 (PMC10515642; doi:10.1021/acs.langmuir.3c01686)
Supplement: Supplementary file 1 — la3c01686_si_001.pdf [file la3c01686_si_001.pdf]

Supporting Information: Liquid–liquid phase separation induced by vapour transfer in evaporative binary sessile droplets

Ahmed M. Othman,[*,†] Andreas. S. Poulos,[‡] Ophelie Torres,[‡] and Alexander. F. Routh[*,†]

†*Department of Chemical Engineering and Biotechnology, University of Cambridge, Philippa Fawcett Dr, Cambridge, CB3 0AS, United Kingdom*

‡*Unilever R&D Port Sunlight, Quarry Road East, Wirral, CH63 3JW, United Kingdom*

E-mail: ao435@cam.ac.uk; afr10@cam.ac.uk

Contents

Octamethyltrisiloxane and ethanol droplets drying images **S-3**

Refractive index calibration curves **S-4**

Octamethyltrisiloxane diffusivity estimation **S-5**

Drying conditions: relative humidity and temperature **S-6**

Octamethyltrisiloxane-Ethanol Droplet Drying Images

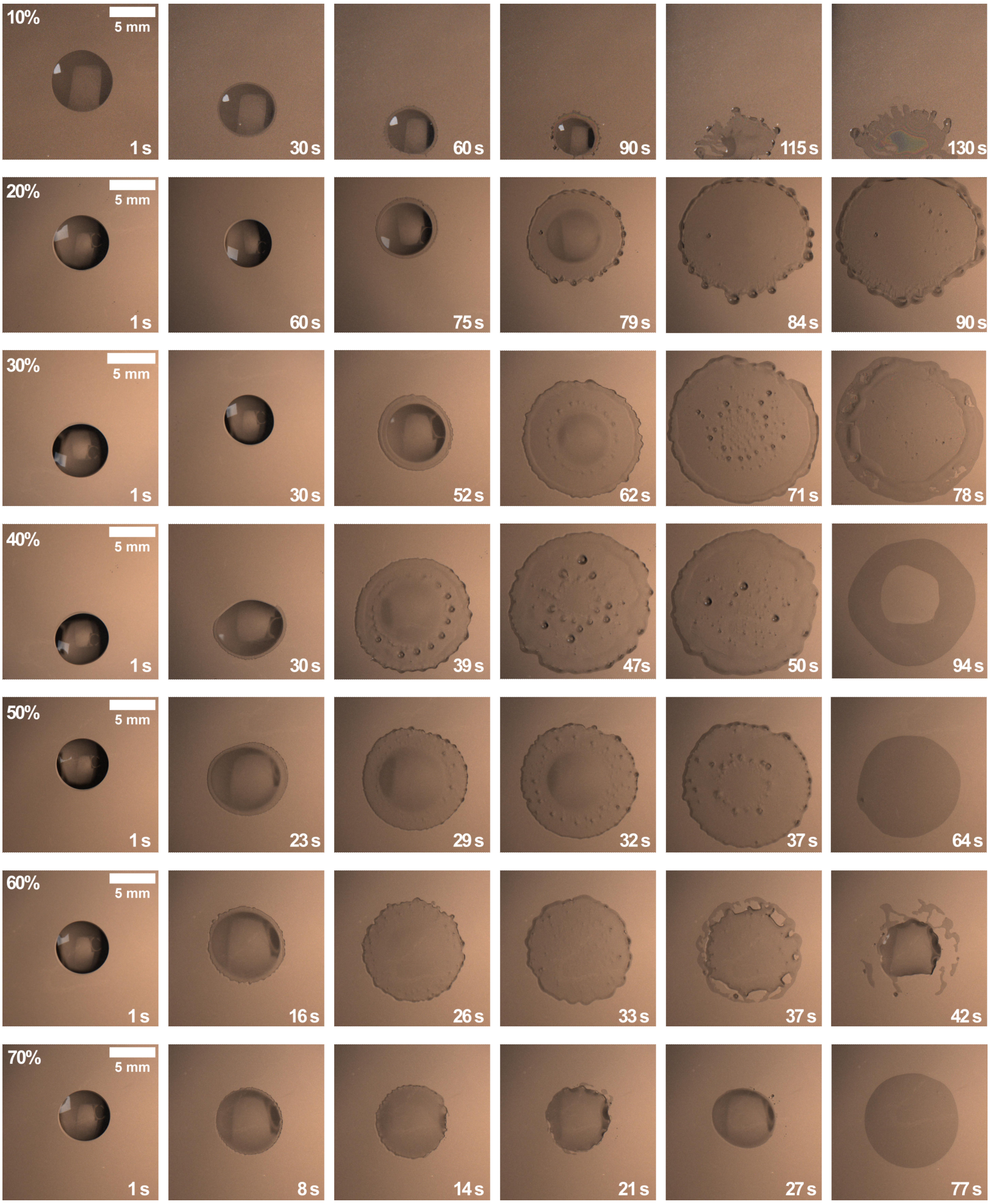

Figure S1: Top-view images of octamethyltrisiloxane and ethanol droplets at different time intervals and initial octamethyltrisiloxane compositions. In each sequence, a 5 μl droplet is deposited onto a pre-cleaned glass slide at 23°C and 49% RH. The droplets are then imaged using a CCD camera. The initial octamethyltrisiloxane concentration of each droplet is highlighted in the top-left corner of each image sequence.

Refractive Index Calibration Curves

Vapour–liquid equilibrium concentrations were found by employing the refractive index after separating both compositions in the pressure custom apparatus, as shown in Figure 1b in the main manuscript. This was achieved by preparing a set of known concentrations of octamethyltrisiloxane and ethanol mixtures. Subsequently, a calibration curve was produced using the corresponding refractive index to determine the measured unknown concentration. The calibration curve, depicting the relationship between refractive index and concentration, is displayed in Figure S2a.

In a similar approach, the quantity of water transported into an ethanol droplet was determined using the refractive index. The corresponding calibration curve, demonstrating the correlation between the refractive index and water content for a combination of ethanol and water, is presented in Figure S2b.

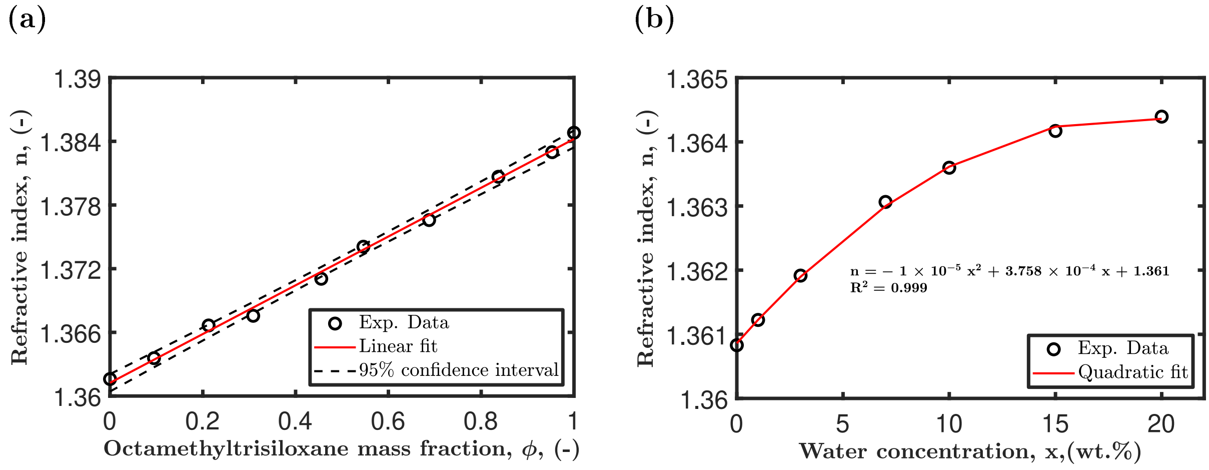

Figure S2: Refractive index as a function of mass fraction calibration curves for (a) octamethyltrisiloxane and ethanol mixture, which was used to determine vapour–liquid equilibrium compositions, and (b) ethanol and water mixture, which was used to determine the amount of water content within the droplet. The refractive index measurements were carried out at 23°C using a Bellingham refractometer.

Octamethyltrisiloxane Diffusivity Estimation

The lack of experimental data on the diffusivity of octamethyltrisiloxane in air in the literature necessitated using an estimation method. To this end, the Fuller-Schettler-Giddings equation was utilised, as it represents the simplest equation available for the prediction of diffusivity for non-polar binary mixtures under low pressure conditions. The Fuller-Schettler-Giddings correlation is expressed as follows:[S1]

$$D_{AB} = \frac{0.001T^{1.75}\left(\frac{1}{M_A} + \frac{1}{M_B}\right)^{1/2}}{P[(\sum v)_A^{1/3} + (\sum v)_B^{1/3}]^2}, \tag{S1}$$

where D_{AB} is the binary gas diffusivity of A in B, T is temperature, M_B, M_B are molecular weights of A and B, P is pressure, and $\sum v_A$, $\sum v_B$ are the sum of molar volumes of components A and B, which are given in Ref. S2. The values used to estimate diffusivity of octamethyltrisiloxane in air are presented in Table S1.

Table S1: Values for estimating diffusion coefficient of octamethyltrisiloxane in air

Parameter	Value
Temperature (T)	296.15 K
Octamethyltrisiloxane molecular weight (MW_A)	236.53 g.mol^{-1}
Air molecular weight (MW_B)	28.1 g.mol^{-1}
Pressure (P)	1 atm
Octamethyltrisiloxane molar volume ($\sum v_A$)	172.36 cm^3.mol^{-1}
Air molar volume ($\sum v_B$)	20.1 cm^3.mol^{-1}

Drying Conditions: Relative Humidity and Temperature

Drying experiments for octamethyltrisiloxane and ethanol droplets were conducted under two controlled relative humidity conditions. The atmospheric relative humidity in the chamber was maintained at an approximate level of 49% RH, as depicted in Figure S3a. In order to reduce the relative humidity further, a saturated potassium hydroxide salt was introduced inside the drying chamber and left to stabilise. Following this, a steady-state value of around 21% RH was achieved after 400 minutes, as shown in Figure S3b.

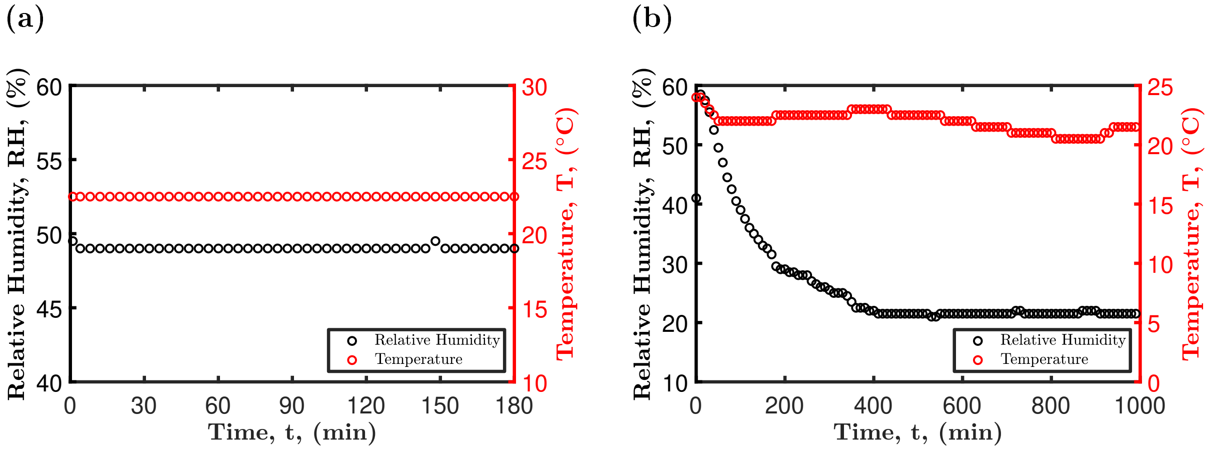

Figure S3: Relative humidity and temperature within the enclosed chamber surrounding the evaporated droplet. (a) At a high relative humidity value of 49% RH, which corresponds to the ambient conditions. (b) At a low relative humidity value of 21% RH, which was obtained by using a saturated potassium hydroxide salt solution.

References

(S1) Welty, J.; Wicks, C.; Wilson, R. E.; Rorrer, G. *Fundamentals Of Momentum, Heat, And Mass Transfer, 5th Ed*; John Wiley & Sons Ltd, 2007; p 445.

(S2) Silcox, G. D.; noble, J. J.; Sarofim, A. F.; Wankat, P. C.; Knaebel, K. S. In *Perry's Chemical Engineers' Handbook*, 9th ed.; Green, D., Southard, M., Eds.; McGraw Hill LLC, 2018; Chapter 5, pp 47–49.
